# Relapsing allergic bronchopulmonary aspergillosis as a trigger for Kounis syndrome: a case report

**DOI:** 10.3389/fcvm.2026.1811823

**Published:** 2026-06-09

**Authors:** Haolei Wei, Liyan Wu, Bin Ni, Jingyu Zhang, Mengxia Xu, Liang Guo, Yanping Mao

**Affiliations:** 1 Department of Intensive Care Medicine, Affiliated Hospital of Hangzhou Normal University, Hangzhou, Zhejiang, China; 2 Xiangcheng Branch, Traditional Chinese Medicine Hospital of Xiaoshan District, Hangzhou, Zhejiang, China; 3 Department of Cardiology, Affiliated Hospital of Hangzhou Normal University, Hangzhou, Zhejiang, China; 4 Department of Intensive Care Medicine, Zhejiang Xiaoshan Hospital, Hangzhou, Zhejiang, China

**Keywords:** acute coronary syndrome, allergic bronchopulmonary aspergillosis, Kounis syndrome, hypersensitivity reaction, case report

## Abstract

Kounis syndrome is an allergic acute coronary syndrome triggered by hypersensitivity reactions. Its association with allergic bronchopulmonary aspergillosis (ABPA) is rarely reported. We describe a 77-year-old woman with asthma, chronic obstructive pulmonary disease, and established ABPA who developed acute chest tightness and dyspnea during an ABPA relapse. Laboratory testing showed marked eosinophilia, elevated total IgE, and evidence of myocardial injury, with increased cardiac troponin I and B-type natriuretic peptide. Electrocardiography revealed ischemic changes, and coronary angiography demonstrated nonobstructive coronary arteries. Based on the clinical and immunologic findings, Kounis syndrome, possibly triggered by ABPA, was diagnosed. The patient improved with antifungal therapy, systemic corticosteroids, coronary vasodilators, and supportive care. This case highlights ABPA relapse as a potential trigger of Kounis syndrome and emphasizes the need for cardiovascular vigilance in patients with severe allergic airway disease.

## Introduction

In 1991, Kounis introduced the concept of allergic acute coronary syndrome to describe acute coronary events triggered by hypersensitivity reactions (1). Kounis syndrome (KS) refers to the occurrence of acute coronary syndrome in the context of allergic, anaphylactic, hypersensitivity, or anaphylactoid reactions (2). Although it is increasingly reported, KS remains poorly recognized and is often underdiagnosed in clinical practice (3). A wide range of triggers has been identified, including foods, environmental exposures, medical conditions, and insect stings; however, pharmacologic agents—particularly antibiotics—are the most common precipitants (4, 5). Clinical manifestations range from coronary vasospasm and chest pain to myocardial infarction and acute heart failure. To date, no consensus statements or standardized clinical guidelines for the management of KS have been established.

KS is classified into three subtypes: type I, characterized by coronary vasospasm in patients with angiographically normal coronary arteries; type II, occurring in individuals with preexisting atherosclerosis and presenting with vasospasm or myocardial infarction; and type III, associated with allergic reactions following coronary stent implantation and in-stent thrombosis (6).

Allergic bronchopulmonary aspergillosis (ABPA) is an immunologically mediated pulmonary hypersensitivity disorder caused by an exaggerated allergic response to inhaled *Aspergillus* spores (7). Pathogenesis involves type I and type III hypersensitivity reactions, leading to the release of inflammatory mediators that promote airway inflammation, mucus impaction, bronchiectasis, and pulmonary consolidation or fibrosis (8, 9). ABPA occurs predominantly in patients with asthma or cystic fibrosis (7, 10). Given shared immunopathological mechanisms between ABPA and KS, systemic allergic responses during ABPA exacerbations may theoretically contribute to coronary vasospasm and myocardial injury consistent with KS.

Here, we report a case of KS that was potentially triggered by ABPA. The patient presented with atypical chest tightness and dyspnoea in the setting of established ABPA, which delayed recognition of cardiac involvement. This case highlights the importance of considering allergic coronary syndromes in patients with ABPA who develop unexplained cardiopulmonary symptoms.

## Case description

### Patient information

A 77-year-old woman was admitted with recurrent cough, sputum production, and exertional dyspnoea for more than 7 years, with acute worsening over 2 days. Medical history included asthma, chronic obstructive pulmonary disease (COPD), and prior surgical resection for right lung and left upper-lobe malignancies. No drug or food allergies were documented.

### Clinical history and diagnostic assessment

Seven years earlier, the patient developed a chronic cough with yellow-white viscous sputum, wheezing, and exertional chest tightness. These symptoms were initially treated as COPD exacerbations, with partial and transient relief. In 2022, the patient was rehospitalized for asthma/COPD and was found to have markedly elevated total immunoglobulin E (IgE; > 1,000 IU/mL), peripheral eosinophilia (> 0.5 × 10^9^/L), high-resolution computed tomography (HRCT) findings of mucus impaction with a “finger-in-glove” pattern, and positive fungal allergen test results, including *Aspergillus fumigatus*. ABPA was diagnosed, and symptoms improved with itraconazole.

### Current presentation

Two days before admission, respiratory symptoms worsened. The eosinophil count was 1.71 × 10^9^/L. Despite bronchodilator therapy, symptoms persisted. HRCT showed bilateral bronchiectasis with inflammatory changes (Figure 1). An ABPA relapse was suspected, and itraconazole and supportive therapy were restarted; eosinophil levels subsequently declined to zero.

**Figure 1:**
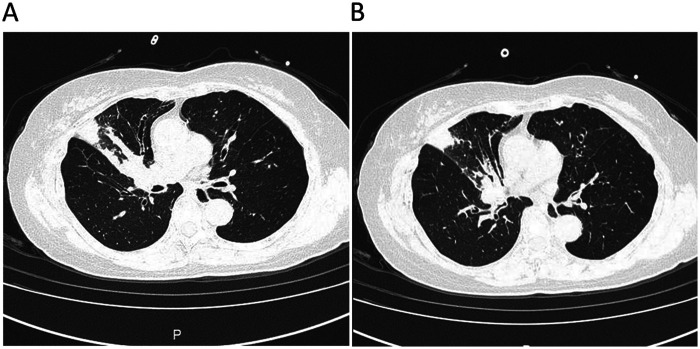
High-resolution chest computed tomography images. **(A, B)** The scans demonstrate bilateral inflammatory changes with fibrotic streaks, multifocal bronchiectasis, and partial bronchial obstruction with consolidation in the right middle lobe. Post-surgical changes are evident in the left lung, accompanied by focal pleural thickening bilaterally.

### Development of cardiac manifestations

The next day, the patient developed sudden chest tightness and dyspnoea. B-type natriuretic peptide (BNP) increased from 132.5 to 1,150.8 pg/mL, and cardiac troponin I (cTnI) was 2.041 ng/mL (Figure 2). Electrocardiography (ECG) showed T-wave inversions in leads V4–V6 and II, III, and aVF (Figure 3). Non–ST-segment elevation acute coronary syndrome was suspected, and the patient was transferred to the intensive care unit (ICU).

**Figure 2:**
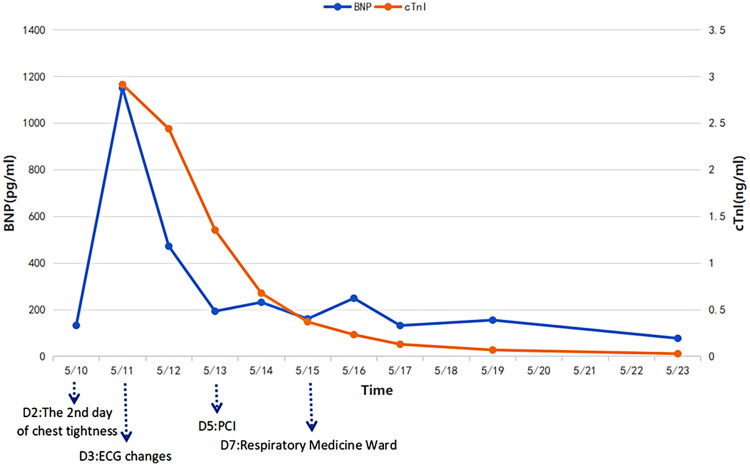
Temporal changes in serum cardiac biomarkers. Serial measurements of cardiac troponin I (cTnI; reference range, 0–0.026 ng/mL; right *y* axis) and B-type natriuretic peptide (BNP; reference range, 0–100 pg/mL; left *y* axis) demonstrate myocardial injury following an acute exacerbation of allergic bronchopulmonary aspergillosis.

**Figure 3:**
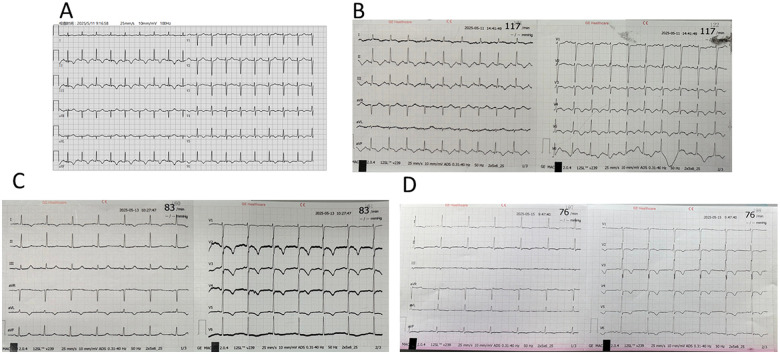
Electrocardiographic findings. **(A)** On Day 3 after symptom onset, electrocardiography demonstrated T-wave inversion in leads V4–V6 and II, III, and aVF, coinciding with the development of chest tightness and dyspnoea. **(B,C)** On the day following transfer to the ICU, persistent T-wave inversion was observed in leads V3–V6 and II, III, and aVF, accompanied by ST-segment depression. **(D)** Six days after the onset of chest tightness and dyspnoea, when the patient had become asymptomatic, the electrocardiogram demonstrated partial resolution of T-wave inversion in leads V3–V6 and II, III, and aVF, with normalization of the ST segment.

On ICU admission (Day 3), vital signs were notable for tachycardia (122 beats/min) and tachypnoea (30/min), with a blood pressure of 151/107 mmHg. Orthopnoea and bilateral moist rales were present. Cardiac troponin I increased to 2.678 ng/mL, with persistent ECG abnormalities. Later that evening, cTnI further rose to a peak of 2.916 ng/mL. Acute coronary syndrome complicated by acute left heart failure was diagnosed. No new medications or contrast agents had been introduced, and itraconazole had been tolerated previously.

### Therapeutic interventions

Initial management consisted of high-flow oxygen alternating with noninvasive ventilation, itraconazole combined with piperacillin–tazobactam, milrinone, isosorbide mononitrate, clopidogrel, low-molecular-weight heparin, and atorvastatin. On Day 4, cTnI remained elevated (2.441 ng/mL), B-type natriuretic peptide (BNP) decreased to 472.7 pg/mL, and total immunoglobulin E (IgE) was 2,148 IU/mL. Echocardiography revealed globally reduced left ventricular ejection fraction (LVEF, 39%), with diffuse left ventricular hypokinesis, most pronounced in the anterior septum and anterior wall, consistent with impaired systolic function.

On Day 5, coronary angiography demonstrated only mild atherosclerosis—approximately 20% stenosis in the proximal left anterior descending artery with distal myocardial bridging and 20%–30% stenosis in the proximal right coronary artery—thereby excluding obstructive coronary disease (Figure 4). Given the temporal association with allergic bronchopulmonary aspergillosis (ABPA) relapse, marked eosinophilia and elevated IgE levels, evidence of myocardial injury in the absence of obstructive coronary lesions, and subsequent improvement following antifungal and corticosteroid therapy, Kounis syndrome (KS) was considered, with ABPA relapse identified as a potential trigger. Mast cells are central to the pathogenesis of KS. Although specific mediators were not measured in this case, the clinical constellation supports the proposed diagnosis. Clopidogrel and atorvastatin were discontinued, while itraconazole, anticoagulation, and systemic corticosteroids (methylprednisolone) were continued.

**Figure 4:**
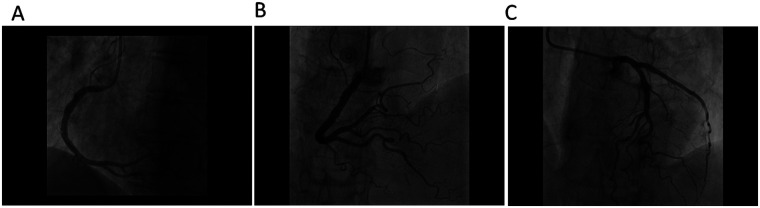
Coronary angiography findings. **(A–C)** Coronary angiography reveals mild atherosclerotic changes in the proximal left anterior descending artery with approximately 20% luminal stenosis. The left main coronary artery, left circumflex artery, and diagonal branches show no significant stenosis. Mild luminal narrowing of approximately 20%–30% is observed in the proximal right coronary artery. No significant stenosis is present in the posterior descending or posterolateral branches.

### Follow-up and outcomes

*Aspergillus fumigatus*–specific IgE and IgG were positive. Cardiopulmonary symptoms improved, and the patient was transferred back to the respiratory ward on Day 7. Repeat echocardiography demonstrated diffuse left ventricular hypokinesis, predominantly affecting the mid-to-apical segments of the interventricular septum and anterior wall, with relatively preserved basal contractility. The LVEF was 47%, indicating partial recovery of systolic function compared with prior assessment. Biomarkers and ECG changes gradually resolved. Anticoagulation was switched to rivaroxaban due to hematuria. Corticosteroids were tapered to oral prednisone. By Day 16, BNP levels had normalized, and cTnI was only minimally elevated (0.029 ng/mL). The patient was discharged on rivaroxaban, sacubitril/valsartan, and itraconazole (Supplementary Figure S1).

## Discussion

The incidence of KS is likely underestimated due to its nonspecific clinical presentation and limited awareness among clinicians. A prospective study conducted in an emergency department reported an incidence of approximately 19.4 cases per 100,000 individuals, accounting for about 3.4% of patients admitted with allergic conditions (11). A wide spectrum of allergens has been implicated, with medications, particularly antibiotics, nonsteroidal anti-inflammatory drugs, and contrast agents, being the most frequent triggers (12, 13), followed by foods and insect stings (14, 15).

Based on established immunopathological mechanisms and the clinical features observed in this patient, an acute hypersensitivity response associated with ABPA may plausibly precipitate acute coronary events. ABPA is characterized by an exaggerated immune response to *Aspergillus* antigens, involving both IgE-mediated type I and IgG-mediated type III hypersensitivity reactions (8, 16). Sensitized CD4⁺ T cells secrete interleukin (IL)-4, IL-5, and IL-13, which promote B-cell differentiation and the production of *Aspergillus*-specific IgE and IgG antibodies (17, 18). Subsequent activation and degranulation of mast cells and eosinophils result in the release of histamine, leukotrienes, prostaglandins, and other inflammatory mediators (19, 20). Clinically, these processes are manifested by marked peripheral eosinophilia, persistent airway inflammation, mucus impaction, and bronchiectasis (19, 21).

Comprehensive allergological testing for antimicrobial agents was not performed in this case. Conventional testing identified sensitization to *Aspergillus* but did not fully exclude a drug-related mechanism. However, several features argue against drug-induced KS. No new medications were introduced prior to symptom onset, and the temporal pattern was not consistent with a drug-triggered hypersensitivity reaction. In contrast, the clinical deterioration occurred in the context of ABPA exacerbation, accompanied by marked eosinophilia, elevated IgE levels, and a favorable response to targeted antifungal and anti-inflammatory therapy, collectively supporting ABPA as the most likely trigger.

From a mechanistic perspective, the association between ABPA exacerbation and acute coronary involvement is likely mediated by systemic immune activation and downstream vascular effects. During acute exacerbations of ABPA, inflammatory mediators may enter the systemic circulation and affect the coronary vasculature, thereby triggering allergic coronary events (6, 22). Histamine can induce coronary vasospasm and myocardial ischemia through direct effects on vascular smooth muscle (23), while leukotrienes may act as potent vasoconstrictors. Mast cell proteases, particularly chymase, may enhance local angiotensin II generation and thereby augment vasoconstrictive responses (23, 24). In addition, histamine facilitates platelet activation and aggregation, thereby increasing the risk of thrombosis (23). These mechanisms are inferred from established literature on ABPA and KS and were not directly measured in this patient; however, they provide a biologically plausible framework linking allergic inflammation to coronary dysfunction. Collectively, these observations are consistent with a mediator-driven and potentially reversible mechanism of myocardial injury.

A notable feature of this case is the apparent temporal dissociation between normalization of peripheral eosinophil counts and the onset of cardiac symptoms. This observation underscores that circulating eosinophil levels may not accurately reflect tissue-level inflammatory activity. During ABPA exacerbation, eosinophils are rapidly recruited into affected tissues, and local activation may persist despite declining peripheral counts following treatment. Moreover, the cardiovascular manifestations of KS are primarily mediated by vasoactive substances, including histamine and leukotrienes, which may be released before or during the decline in circulating eosinophils. These mediators can exert delayed effects on the coronary vasculature, resulting in a lag between systemic immune activation and clinical cardiac presentation. In addition, mast cell activation—central to KS—is not captured by routine blood indices and may persist independently of eosinophil dynamics. Accordingly, normalization of peripheral eosinophils should not be interpreted as resolution of the underlying allergic process.

Although KS was initially diagnosed, coronary angiography revealed only mild, non-obstructive coronary artery disease. The distinction between type I and type II KS remains challenging in such cases. Type II KS is defined by the occurrence of allergic coronary events in the presence of pre-existing atherosclerosis; however, the degree of stenosis required for this classification is not clearly established (6, 25, 26). In the present case, the absence of plaque rupture or thrombosis, together with minimal luminal narrowing, suggests that both type I (vasospasm in near-normal arteries) and type II mechanisms may coexist. Therefore, this case is more appropriately interpreted along a pathophysiological spectrum rather than within a rigid subtype classification.

Takotsubo cardiomyopathy (TCM) represents an important differential diagnosis. ABPA exacerbation constitutes a significant physiological stressor and could theoretically precipitate TCM, particularly in the presence of elevated cardiac biomarkers and reduced left ventricular ejection fraction. However, several features argue against classic TCM. The cardiac event demonstrated a clear temporal association with allergic inflammation, accompanied by marked eosinophilia and elevated IgE levels. Furthermore, echocardiography revealed diffuse left ventricular dysfunction, predominantly involving the interventricular septum and anterior wall, rather than the characteristic apical or mid-ventricular ballooning pattern. Nevertheless, atypical TCM cannot be definitively excluded because cardiac magnetic resonance imaging was not performed and complete longitudinal documentation of ventricular functional recovery was unavailable.

Eosinophilic myocarditis also warrants consideration, as it may present with elevated cardiac biomarkers and non-obstructive coronary arteries in the setting of systemic eosinophilia. However, eosinophilic myocarditis is typically characterized by myocardial infiltration, necrosis, and a more sustained or progressive course. In contrast, the present case demonstrated rapid clinical and functional recovery following antifungal and corticosteroid therapy, without the need for additional immunosuppression. This pattern is more consistent with transient, mediator-driven myocardial dysfunction than with primary myocardial inflammation. Nevertheless, definitive differentiation would require advanced imaging or histological confirmation. Cardiac magnetic resonance imaging with late gadolinium enhancement, or endomyocardial biopsy, was not performed in this case and represents a limitation. Therefore, the diagnosis of KS should be considered clinically supported but not definitive. In addition, serum tryptase was not measured, precluding direct assessment of mast cell activation.

Currently, no standardized treatment guidelines exist for KS. Management generally involves a combination of conventional acute coronary syndrome therapy and targeted anti-allergic treatment, with prompt identification and control of the underlying trigger being essential (6, 27). In the present case, effective management of ABPA was central to clinical stabilization. The patient's initial ABPA episode responded to itraconazole monotherapy; however, relapse with new pulmonary infiltrates supported the initiation of combined systemic corticosteroids and antifungal therapy, which likely contributed to the resolution of the associated coronary manifestations. Current recommendations suggest an acute-phase prednisone dose of 0.5 mg·kg^−1^·day^−1^, followed by gradual tapering after 2–4 weeks, for a total duration of approximately four months, in combination with itraconazole 200 mg twice daily for the same duration (28).

Although KS occurs in the setting of allergic or hypersensitivity reactions, epinephrine and antihistamines were not administered in this patient. This decision reflected the clinical context, in which the presumed trigger was ABPA exacerbation and management therefore focused primarily on controlling the underlying type 2 inflammatory response and reducing fungal burden. Although epinephrine remains the first-line treatment for systemic anaphylaxis, it was not indicated here because the patient showed no evidence of anaphylactic shock, hemodynamic instability, or other clinical criteria for anaphylaxis. In addition, in suspected KS, epinephrine use may require careful individualization because of its potential to increase myocardial oxygen demand and aggravate coronary vasospasm. Antihistamines were also withheld because the patient did not exhibit urticaria, angioedema, or other prominent histamine-mediated systemic manifestations. In this setting, antihistamines were considered adjunctive rather than essential.

The patient's symptoms were relieved after antifungal therapy and glucocorticoid treatment. Nevertheless, guidelines indicate that IL-5-targeted biologics may be beneficial in the management of ABPA (28). As discussed above, IL-5 plays a pivotal role in the maturation, activation, and survival of eosinophils. The marked eosinophilia and elevated IgE levels observed in this case are typical immunological manifestations of ABPA. By targeting IL-5 or its receptors, anti-IL-5 monoclonal antibodies can effectively reduce eosinophil numbers and activity, thereby alleviating airway inflammation and improving pulmonary function. Given that the onset of Kounis syndrome is closely associated with systemic allergic reactions and the release of inflammatory mediators, targeted interventions targeting inflammatory pathways in ABPA, especially eosinophil-mediated inflammation, may help reduce the risk of Kounis syndrome. A meta-analysis of biologics for ABPA treatment enrolled 86 studies involving 346 patients and demonstrated that omalizumab, dupilumab, and mepolizumab could significantly lower the exacerbation rate and total serum IgE levels, while benralizumab showed a similar trend but did not reach statistical significance (29). According to clinical guidelines for ABPA diagnosis and treatment, treatment-dependent ABPA is defined by any of the following criteria: two or more recurrent acute exacerbations of ABPA occurring within 3 months after glucocorticoid withdrawal; aggravation of respiratory symptoms accompanied by imaging deterioration or a 50% increase in total serum IgE within 4 weeks of oral steroids tapering on two separate occasions. Current guidelines only recommend biologics for treatment-dependent ABPA rather than as first-line therapy for acute ABPA (28). Therefore, given the patient's financial status, biologics were not administered in this clinical case. However, given the core role of IL-5 in the inflammatory cascade of ABPA and the potential involvement of eosinophils in the pathogenesis of Kounis syndrome, such precision medicine strategies can provide more optimized therapeutic options for ABPA patients when accessible and may further prevent severe complications, including Kounis syndrome.

Management of the acute coronary syndrome required therapeutic reassessment. The patient was initially treated for non–ST-segment elevation acute coronary syndrome with clopidogrel and atorvastatin. However, after coronary angiography excluded significant obstructive coronary disease, the treatment strategy was reconsidered. Given that angiography demonstrated only mild, non-obstructive atherosclerosis, the acute coronary event was considered more likely to reflect a functional mechanism, such as coronary vasospasm or microvascular dysfunction, rather than plaque rupture or thrombosis. In this context, the benefits of continued antiplatelet and statin therapy were uncertain, whereas the potential risks, particularly bleeding risk in an elderly patient, remained clinically relevant. Following multidisciplinary discussion, antiplatelet and statin therapy were therefore temporarily discontinued, and treatment was redirected toward control of the underlying allergic trigger and coronary vasomotor dysfunction. This approach reflects a mechanism-guided management strategy rather than one based strictly on KS subtype classification.

Before discharge, B-type natriuretic peptide had normalized, whereas troponin remained mildly elevated. Repeat echocardiography showed partial recovery of left ventricular systolic function, with the left ventricular ejection fraction improving to 47% but not yet returning to normal. These findings suggested residual, albeit improving, myocardial dysfunction. On this basis, sacubitril/valsartan was prescribed as short-term, transitional supportive therapy during recovery rather than as definitive long-term heart failure treatment. As left ventricular systolic function had not yet fully normalized at discharge (LVEF 47%), continued pharmacologic support was considered appropriate. Ongoing follow-up was planned, with treatment discontinuation to be considered after complete functional recovery.

The patient was assessed as being at high risk for venous thromboembolism (VTE) on ICU admission, with a VTE score of 8, and prophylactic low-molecular-weight heparin was initiated. After transfer to the respiratory ward, hematuria developed and resolved following treatment discontinuation, suggesting a possible association with heparin exposure. After cardiology consultation, thromboprophylaxis was switched to rivaroxaban. Consensus recommendations support individualized consideration of extended thromboprophylaxis for up to 30 days in selected high-risk patients without major bleeding risk factors, using either low-molecular-weight heparin or rivaroxaban. Because the patient remained temporarily immobile after discharge, short-term extended thromboprophylaxis was considered appropriate, with planned reassessment and discontinuation once mobility recovered. Importantly, rivaroxaban was prescribed solely for thromboprophylaxis related to elevated VTE risk and transient immobility, rather than as treatment for KS or obstructive coronary artery disease.

Overall, this case highlights a potential gap in cardiovascular risk assessment among patients with ABPA. Marked eosinophilia and elevated total serum IgE are hallmarks of ABPA-associated allergic inflammation. Clinicians should therefore consider cardiovascular evaluation—including cardiac biomarkers and electrocardiography—when patients with ABPA present with atypical chest discomfort or unexplained dyspnea, even in the absence of classic anginal symptoms. Although a single case cannot establish causality, a drug-induced mechanism appears unlikely given the absence of newly introduced medications and the patient's prior tolerance to itraconazole, supporting ABPA relapse as the most plausible trigger.

## Conclusion

To our knowledge, this case represents one of the first reports describing KS in temporal association with a relapse of ABPA, thereby contributing to the limited literature on the potential etiological contexts of this condition. It highlights the importance of increased clinical awareness regarding the interaction between allergic inflammation and cardiovascular events in patients with ABPA. When cardiac symptoms develop during acute allergic episodes, KS should be considered in the differential diagnosis. Further studies are warranted to elucidate the underlying mechanisms and enhance early recognition of these cross-system manifestations, thereby reducing missed diagnoses and improving patient outcomes.

## Data Availability

The original contributions presented in the study are included in the article/Supplementary Material, further inquiries can be directed to the corresponding authors.

## Supplementary material

Supplementary Figure S1 Clinical timeline of ABPA exacerbation associated with suspected Kounis syndrome and subsequent recovery. HRCT, high-resolution computed tomography; ECG, electrocardiography; LVEF, left ventricular ejection fraction.
