# Supplementary material for: Relapsing allergic bronchopulmonary aspergillosis as a trigger for Kounis syndrome: a case report
Source: Front Cardiovasc Med. 2026 Jun 9;13:1811823. doi: 10.3389/fcvm.2026.1811823 (PMC13288271; doi:10.3389/fcvm.2026.1811823)
Supplement: Supplementary file 1 [file Supplementaryfile1.pdf]

知情同意书

同意声明

1. 本研究及论文发表项目相关责任人已向本人完整、详细解释本次病例报告发表的研究目的、研究内容、发表用途、传播范围及相关学术意义，本人已完全知晓全部内容。

The relevant personnel in charge of this research and paper publication have fully and elaborately explained the research purpose, content, publication purpose, dissemination scope and relevant academic significance of this case report to me, and I have fully understood all the contents.

2. 本人已就本病例报告发表相关问题向项目责任人进行充分咨询、提问与沟通，所有疑问均得到清晰、专业的解答，本人对解答结果完全满意，无任何未尽疑问。

I have fully consulted, questioned and communicated with the relevant personnel in charge of this research, on issues related to the publication of this case report. All questions have been answered clearly and professionally. I am completely satisfied with the answers and have no remaining doubts.

3. 我有充足的时间作出决定。

I have sufficient time to make decision.

4. 本人确认完全自愿、自主同意将本人病例资料用于本次病例报告及论文发表。

I confirm that I voluntarily and independently agree to use my case data for this case report and paper publication.

5. 本人已完整阅读、核对本次待发表病例报告的全部文章内容，确认文章中涉及本人病情、诊疗过程的相关描述真实、准确，无虚假、篡改及不实表述。

I have completely read and checked all the contents of this to-be-published case report, and confirm that the relevant descriptions of my condition and diagnosis and treatment process in the article are true and accurate, without falsehood, tampering or untrue statements.

6. 本人知晓，项目方将对本人所有个人隐私信息（姓名、联系方式、家庭住址等）进行完全脱敏处理，论文发表及传播过程中不会泄露本人任何可识别个人身份的信息，充分保障本人隐私权。

I acknowledge that the project team will completely desensitize all my personal privacy information (name, contact information, home address, etc.), and will not disclose any personally identifiable information during the publication and dissemination of the paper, so as to fully

protect my right of privacy.

7. 本人知晓，本次病例报告发表为纯学术公益交流，无任何商业收益，本人与本研究相关责任人、医院间无经济权益纠纷。

I acknowledge that the publication of this case report is a pure academic non-profit communication without any commercial benefits. There are no economic disputes between me, the relevant personnel responsible for this research, and the hospital.

8. 我将获得一份经过签名并注明日期的知情同意书副本。

I will receive a signed and dated copy of this informed consent.

本人充分理解以上所有条款，同意项目负责人完成本次病例报告的投稿与公开发表工作。

I fully understand all the above clauses and agree that the relevant personnel in charge of this research completes the submission and public publication of this case report.

研究参与者签名：[illegible]　　　　日期：2026 年 6 月 7 日

研究参与者联系电话：13456918202

法定代表人签字：　　　　与研究参与者关系：

法定代表人联系电话：　　　　日期：　年　月　日

（研究参与者如为未成年人，则必须要法定代表人签字）：

我确认已向研究参与者/法定代表人解释了本研究的详细情况，包括其权利以及可能的受益和风险，并给其一份签署过的知情同意书副本。

医生签名：[illegible]　　　　日期：2026 年 6 月 7 日

研究医生联系方式：18967162156

（此页为研究参与者知情同意书的必要部分，每一份“研究参与者知情同意书”必须有研究参与者或法定代理人及研究医生的签字和日期，方为有效。）
