# Supplementary figures and images for: Relapsing allergic bronchopulmonary aspergillosis as a trigger for Kounis syndrome: a case report
Source: Front Cardiovasc Med. 2026 Jun 9;13:1811823. doi: 10.3389/fcvm.2026.1811823 (PMC13288271; doi:10.3389/fcvm.2026.1811823)

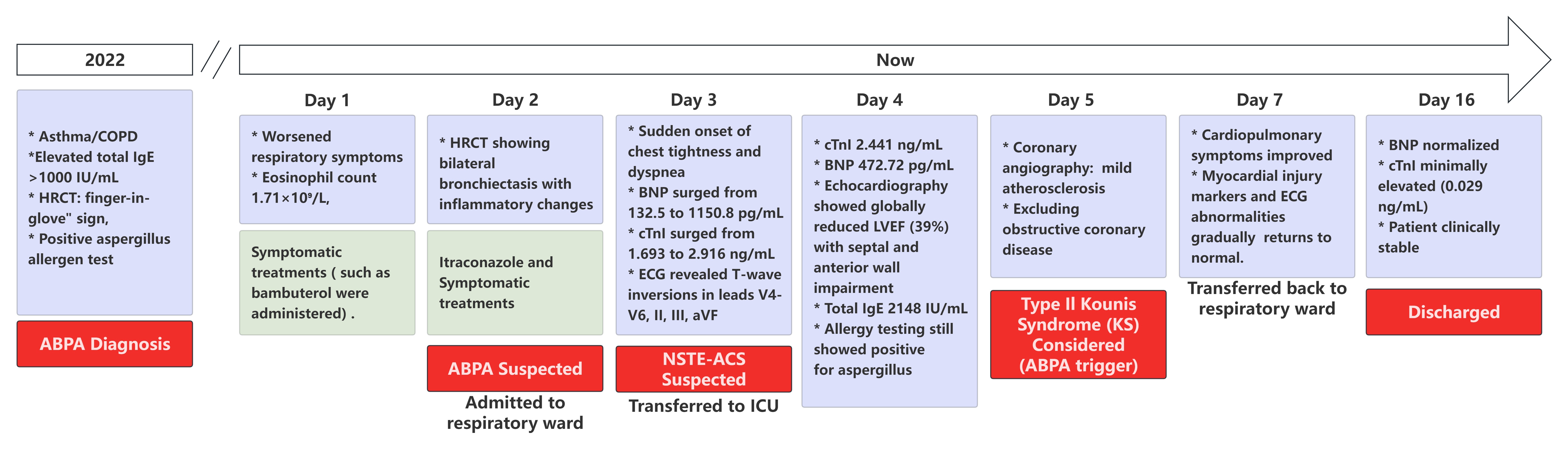

Supplement: Supplementary Figure S1 — Clinical timeline of ABPA exacerbation associated with suspected Kounis syndrome and subsequent recovery. HRCT, high-resolution computed tomography; ECG, electrocardiography; LVEF, left ventricular ejection fraction. [file Image1.jpeg]
